# Oral squamous cell carcinomas: state of the field and emerging directions

**DOI:** 10.1038/s41368-023-00249-w

**Published:** 2023-09-22

**Authors:** Yunhan Tan, Zhihan Wang, Mengtong Xu, Bowen Li, Zhao Huang, Siyuan Qin, Edouard C. Nice, Jing Tang, Canhua Huang

**Affiliations:** 1 State Key Laboratory of Biotherapy and Cancer Center, West China Hospital, and West China School of Basic Medical Sciences & Forensic Medicine, Sichuan University, and Collaborative Innovation Center for Biotherapy, Chengdu, China; 2 West China Hospital of Stomatology, Sichuan University, Chengdu, China; 3 Department of Biochemistry and Molecular Biology, Monash University, Clayton, VIC Australia; 4 Department of Radiology, West China Hospital, Sichuan University, Chengdu, China

**Keywords:** Cancer, Oral cancer

## Abstract

Oral squamous cell carcinoma (OSCC) develops on the mucosal epithelium of the oral cavity. It accounts for approximately 90% of oral malignancies and impairs appearance, pronunciation, swallowing, and flavor perception. In 2020, 377,713 OSCC cases were reported globally. According to the Global Cancer Observatory (GCO), the incidence of OSCC will rise by approximately 40% by 2040, accompanied by a growth in mortality. Persistent exposure to various risk factors, including tobacco, alcohol, betel quid (BQ), and human papillomavirus (HPV), will lead to the development of oral potentially malignant disorders (OPMDs), which are oral mucosal lesions with an increased risk of developing into OSCC. Complex and multifactorial, the oncogenesis process involves genetic alteration, epigenetic modification, and a dysregulated tumor microenvironment. Although various therapeutic interventions, such as chemotherapy, radiation, immunotherapy, and nanomedicine, have been proposed to prevent or treat OSCC and OPMDs, understanding the mechanism of malignancies will facilitate the identification of therapeutic and prognostic factors, thereby improving the efficacy of treatment for OSCC patients. This review summarizes the mechanisms involved in OSCC. Moreover, the current therapeutic interventions and prognostic methods for OSCC and OPMDs are discussed to facilitate comprehension and provide several prospective outlooks for the fields.

## Introduction

Oral squamous cell carcinoma (OSCC), which develops in the oral mucosa, is a common type of head and neck malignancy.^1–3^ According to data collected by the Global Cancer Observatory (GCO), there were 377,713 cases of OSCC worldwide in 2020, with the majority occurring in Asia.^4^ OSCC affects more males than females, with middle-aged to elderly men being the most susceptible.^5^ OSCC results in disfiguration and functional impairments, including swallowing, speech, and taste, which have a substantial impact on the life quality of patients.^6,7^

Clinically, OSCC is characterized by a red and white or red lesion with a slightly uneven surface and distinct borders.^8,9^ Early-stage lesions are typically painless,^10^ but they may cause discomfort and exhibit features such as ulceration, nodularity, and tissue attachment as they progress.^11^ Ulceration is a typical symptom of OSCC, which appears with an irregular floor and margins and is hard upon palpation.^11,12^ The posterior lateral border of the tongue has the highest incidence of OSCC, accounting for an estimated 50% of all OSCC cases,^13^ followed by the mouth floor, the soft palate, the gingiva, the buccal mucosa, and the hard palate.^14^ OSCC spreads predominantly to ipsilateral lymph nodes of the neck via lymphatic outflow, but can also invade contralateral or bilateral lymph nodes. Lungs, bones, and the liver are typical locations for OSCC metastases.^15^

Patients with oral potentially malignant disorders (OPMDs) are more likely than those with healthy mucosa to develop invasive oral carcinomas.^16–20^ At the time of diagnosis, the majority of patients with OPMDs are asymptomatic;^21^ however, some patients may exhibit symptoms of suspected malignancy, such as erythema, pain, tingling sensations, or ulceration.^16^ Consequently, the diagnosis of OPMDs is a crucial method for clinicians to evaluate the risk of OSCC and guide appropriate treatments (Table 1). OPMDs include oral leukoplakia (OL), oral erythroplakia (OE), oral submucosal fibrosis (OSMF), and oral lichen planus (OLP).^22,23^

**Table 1:** The risks factors, characteristics, epidemiology, and diagnosis of OPMDs

OPMDs	Risk factors	Histopathology	Epidemiology	Diagnosis
Homogeneous OL	▪ Tobacco ▪ Alcohol ▪ BQ ▪ HPV	▪ Superficial surface ▪ White surface ▪ Flat surface ▪ Sharp boundaries	▪ Men aged over 40 ▪ Women non-smoking	▪ Biopsy ▪ Toluidine blue ▪ Salivary diagnostics ▪ Brush biopsy
Non-homogeneous OL		▪ Speckled red lesions ▪ Irregular white lesions ▪ Corrugated epidermis ▪ Wrinkled epidermis ▪ Verrucous surface ▪ Exophytic growth ▪ Nodular outgrowths ▪ Polypoid outgrowths	▪ Men aged 50-70	▪ Biopsy ▪ Toluidine blue ▪ Salivary diagnostics ▪ Brush biopsy
PVL		▪ Verrucous surface ▪ Keratotic surface ▪ Multifocal	▪ Women aged over 60	▪ Biopsy
OE	▪ Tobacco ▪ Alcohol ▪ HPV	▪ Fiery red patch ▪ Smooth surface ▪ Velvety surface ▪ Atrophic epithelium ▪ Thin epithelium ▪ Tough texture (Speckled) ▪ Granular texture (Speckled)	▪ People susceptible to HPV	▪ By exclusion
OSMF	▪ BQ ▪ Areca nuts	▪ Vesicles ▪ Blanching mucosa ▪ Fibrosis ▪ Diffuse boundaries	▪ People aged 20–50	▪ Solid biopsy ▪ Liquid biopsy
Reticular OLP	▪ Psychological stress ▪ Medications ▪ Dental materials ▪ EBV	▪ Wickham striae ▪ Hyperkeratotic plaques	▪ Women aged over 40	▪ Direct immunofluorescence
Erosive OLP		▪ Atrophic ulcers ▪ White striae ▪ Keratinization		
Erythematous OLP		▪ Atrophic mucosa		
Plaque-like OLP		▪ White lesions ▪ Hyperkeratotic surface		
Bullous OLP		▪ Bullae ▪ Ulcerative surface		

The World Health Organization (WHO) Collaborating Center defines OL as a permanent, white, and non-scrapable lesion that appears to be “a predominantly white plaque of questionable risk having excluded (other) known diseases or disorders that carry no increased risk for cancer”.^24–27^ OL patients have a prospective risk of malignancy ranging between 1% and 30%.^28^ OL could present as homogeneous or non-homogeneous depending on the color and the texture of the surface.^29^ In non-homogeneous OLs, malignant transformation is more prevalent. Proliferative verrucous leukoplakia (PVL) is a rare form of multifocal OL. PVL plaque has a verrucous and keratotic surface and is asymptomatic and non-homogeneous. PVL exhibits invasive behavior and recurrence following excision,^30^ of which 60% to 100% develop into oral carcinomas.^31^ In addition to PVL, OE displays an elevated potential for malignant transformation, with approximately 50% of patients at risk of progressing to dysplasia, cancer in situ, or aggressive cancer.^32^

OE is an isolated condition described as a “predominantly fiery red patch that cannot be characterized clinically or pathologically as any definable disease”.^33–35^ 85%–90% of early OSCC manifests initially as OE.^36^

OSMF is characterized by burning sensations or intolerance to spicy food, as well as the presence of vesicles on the palate. Histopathological features of OSMF include alterations in epithelial cell morphology and changes in the composition and structure of the connective tissue.^37–39^ OSMF has a 7.6% rate of malignant transformation over 17 years and may be accompanied by OL and other potentially malignant lesions and carcinomas.^40^

OLP is an inflammatory mucocutaneous disorder affecting between 1% and 2% of the general population, of which 0.07% to 5.8% undergo malignant transformation.^41,42^ There are three clinical subtypes of OLP: erythematous or atrophic, reticular, and erosive. Erosive OLP is the most prevalent clinical subtype associated with malignant transformation.^43–45^

**Fig. 1:**
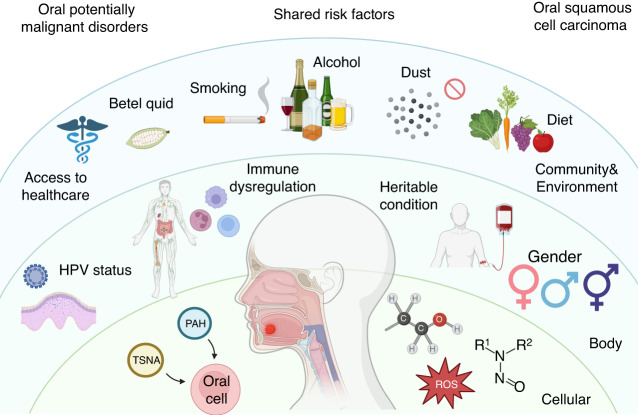
Risk factors of OPMDs and OSCC. The initiation and development of OPMDs and OSCC share similar risk factors, including smoking, alcohol abuse, betel quid (BQ) chewing, human papillomavirus (HPV) infection, nutritional insufficiency, immune deficiency, and hereditary conditions. OPMDs oral potentially malignant disorders, OSCC oral squamous cell carcinoma, PAH polycyclic aromatic hydrocarbons, ROS reactive oxygen species, TSNA tobacco-specific nitrosamines

OPMD and OSCC have complex etiologies, including smoking,^46,47^ alcohol abuse,^48–50^ betel quid (BQ) chewing,^51,52^ human papillomavirus (HPV) infection,^53,54^ nutritional insufficiency,^55^ immune deficiency,^56^ and hereditary conditions (Fig. 1).^57^ The carcinogenicity of polycyclic aromatic hydrocarbons (PAH) and tobacco-specific nitrosamines (TSNA) in tobacco,^58^ ethanol in alcohol,^59^ and nitrosamines in BQ has been demonstrated.^52,60^ Additionally, exposure to dust and heavy metals can cause chronic inflammation or serve as carriers for other oncogenic compounds, thereby increasing the incidence of oral cancer.^61,62^ HPV can cause precancerous squamous intraepithelial neoplasia, which has the potential to become malignant,^63^ and has been hypothesized to assist in OSCC progression.^64^ Nutritional insufficiency, particularly in plant foods and vitamin D, is also related to an elevated potential of oral carcinomas.^65^ Individuals with suppressed immune systems and rare hereditary diseases, such as Fanconi anemia (FA) and dyskeratosis congenital (DC), are more susceptible to OSCC than those with normal physiological function.^66^

Persistent exposure to these risk factors results in genetic alterations, epigenetic modifications, and a dysregulated tumor microenvironment, all of which contribute to the occurrence and transformation of OPMDs to OSCC. The genetic alterations result in the aberrant activation of oncogenic pathways, such as EGFR,^67^ Wnt/β-catenin,^68^ JAK/STAT,^69^ NOTCH,^70^ PI3K/AKT/mTOR,^71^ MET,^72^ and RAS/RAF/MAPK, as well as disruptions of suppressor pathways, such as TP53/RB,^73^ p16/Cyclin D1/Rb,^74^ which significantly contribute to the progression of OSCC. Epigenetic modifications, such as DNA methylation,^75^ histone covalent modification,^76^ chromatin remodeling,^77^ and gene regulation by non-coding RNAs (ncRNAs),^78^ participate in OSCC formation and development. In addition, immune suppression, stromal alteration, hypoxia, and an imbalanced oral microbiome can contribute to the dysregulated tumor microenvironment, thus facilitating OSCC progression.^79–81^

As mentioned above, OSCC may be induced by various risk factors. Chronic exposure to these stimuli promotes carcinogenesis and cancer metastasis by causing genetic mutations, altered epigenetic modification, and a dysregulated tumor microenvironment. Here, we briefly review the mechanisms involved in the occurrence of OSCC. We also discuss therapeutic interventions and the clinical prognosis of OSCC and OPMDs, followed by perspectives for future advancements in the field.

## Genetic alterations drive the occurrence of OSCC

**Fig. 2:**
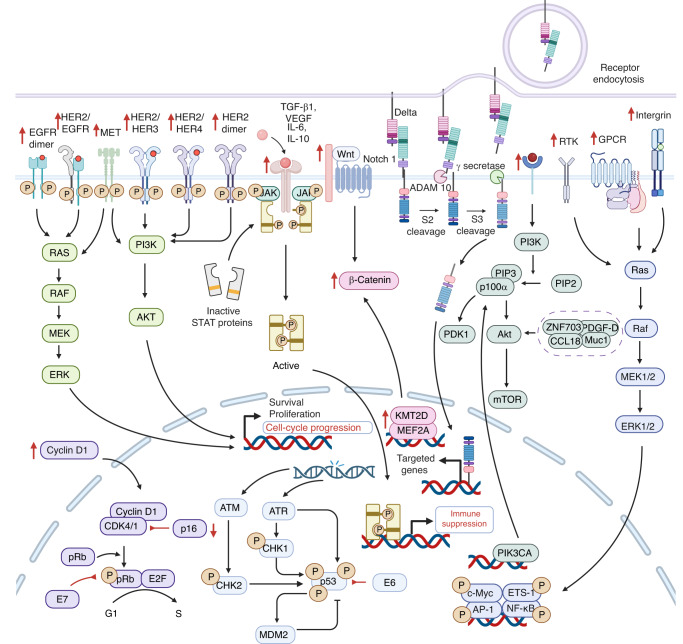
Genetic alterations in OSCC. Genetic alteration in the TP53/RB, p16/Cyclin D1/Rb, EGFR, Wnt/β-catenin, JAK/STAT, NOTCH, PI3K/AKT/mTOR, MET, RAS/RAF/MASK signaling pathways contribute to OSCC progression. EGFR epidermal growth factor receptors, JAK Janus-activated kinase, MAPK mitogen-activated protein kinase, OSCC oral squamous cell carcinoma, RB retinoblastoma, Rb retinoblastoma tumor suppressor protein, STAT signal transducer and activator of the transcription, TP53 tumor protein p53

There exist a multitude of risk factors that have been identified as being capable of inducing genomic alterations, which are commonly observed in both OSCC and OPMDs.^82,83^ Genetic mutations contribute to aberrant activation of oncogenic signaling and inactivation of suppressor signaling, promoting the transformation and uncontrolled proliferation of OSCC cells (Fig. 2).^70,84–86^

### Aberrant activation of oncogenic signaling

Oncogenic signaling pathways, including the EGFR pathway, PI3K/AKT/mTOR pathway, JAK/STAT pathway, MET pathway, Wnt/β-catenin pathway, and RAS/RAF/MAPK pathway, are aberrantly activated and upregulated to promote the progression of OSCC.^87^

#### EGFR pathway

80%-90% of head and neck squamous cell carcinoma (HNSCC) is found to overexpress epidermal growth factor receptors (EGFR), a member of the HER/ErbB family of receptor tyrosine kinases (RTKs).^88–90^ It has been reported that OSCC shows increased EGFR (42% to 58%),^91^ which is associated with poor treatment outcomes and prognosis.^92^

The EGFR pathway prompts OSCC cell proliferation, metastasis, invasion, and apoptosis resistance.^93^ Radiation triggers the translocation of EGFR into the nucleus, where it functions as a transcription factor and leads to radiotherapy resistance in oral cancer.^94^ The EGFR also interacts with other receptors, such as Axl, increasing its carcinogenic potential on the mucosal surface of the oral cavity.^95^

Meanwhile, various factors are implicated in the EGFR pathway to drive the malignancy of OSCC. For instance, downregulated hsa_circ_0005379 facilitates the proliferation and metastasis of OSCC cells by regulating the EGFR pathway.^96^ The overexpression of distal-less homeobox 6 (DLX6) enhances proliferation and inhibits apoptosis in OSCC cells through the EGFR-CCND1 axis.^97^ Upregulated bone marrow stromal cell antigen 2 (BST2) promotes tumor growth and confers gefitinib resistance in OSCC patients via activating the EGFR pathway.^98^

#### PI3K/AKT/mTOR pathway

Thirty-seven percent of HNSCC cases, more specifically 34% of HPV^-^ and 56% of HPV^+^ patients, exhibit overexpression or mutation of PIK3CA, as reported by the Cancer Genome Atlas (TCGA) study.^99,100^ Furthermore, patients with OSCC are more likely to exhibit somatic copy number alterations in genes encoding components of the PI3K/AKT/mTOR network.^71,101–103^

The PI3K/AKT/mTOR pathway leads to the metastasis and proliferation of OSCC cells.^104,105^ The PI3K-AKT pathway is frequently activated in OSCC malignancies due to the evaluated phosphorylation levels of AKT and associated mTOR. Then, by stimulating AKT, PDK1, and mTOR, a cascade of downstream biological processes, such as cell metabolism, cell proliferation, cell death, protein synthesis, and transcription, are increased to drive OSCC.^106^ In the meantime, extracellular ATP stimulates the PI3K-AKT pathway through the P2Y2-Src-EGFR axis to prompt OSCC cell metastasis.^107^ Also, the circEPSTI1/miR-942-5p/LTBP2 axis phosphorylates the components of the PI3K-AKT-mTOR pathway and facilitates epithelial–mesenchyme transition (EMT) to accelerate the metastasis and proliferation of OSCC cells.^108^ Moreover, ITGB2 high cancer-associated fibroblasts (CAFs) stimulate the PI3K-AKT-mTOR pathway to promote the progression of OSCC malignancy via NADH oxidation.^109^ Additionally, a variety of factors, including ZNF703,^110^ PDGF-D,^111^ CCL18,^112^ and Muc1,^113^ activate the PI3K/AKT/mTOR pathway, resulting in OSCC cell survival, invasion, and drug resistance.^105,114^ Collectively, targeting the PI3K/AKT/mTOR pathway could be a potent method to prevent OSCC.

#### JAK/STAT pathway

Both HPV^+^ and HPV^-^ HNSCC display abnormal activation of the signal transducer and activator of the transcription (STAT) pathway.^69,115^ Upregulated STAT3 is associated with HNSCC malignancies and resistance to chemotherapy, radiotherapy, and EGFR-targeted therapy.^116,117^ The STAT3 signaling pathway causes immune suppression and protects OSCC cells from being recognized and destroyed by cytotoxic T cells by stimulating the release of cytokines, such as transforming growth factor (TGF)-β1, vascular endothelial growth factor (VEGF), interleukin (IL)-6, and IL-10.^118^ Moreover, in response to upstream signals from the IL-6 receptor family and RTKs including EGFR, VEGFRs, Jenus-activated kinases (JAKs), and Src family kinases (SFKs), STAT3 is activated and translocated to the nucleus,^119^ thereby inducing the expression of cyclin D1, Bcl-xL, and other pro-survival factors.^118^ In addition, various factors, such as miR-548d-3p and long non-coding RNA (lncRNA) P4713, participate in the JAK/STAT pathway. Specifically, miR-548d-3p binds to the 3’UTR of SOCS5 and SOCS6 to downregulate their expression, regulating the JAK/STAT pathway and serving as an oncogene in OSCC.^120^ lncRNA P4713 activates the JAK/STAT pathway and drives the metastasis and proliferation of OSCC cells.^121^

#### MET pathway

Mutations and gene amplifications in hepatocyte growth factor (HGF) receptor (MET or c-Met) and its ligand HGF are uncommon, occurring in 6% and 2%-13% of HNSCC, respectively.^122–125^ Immunohistochemistry analysis reveals MET and/or HGF are upregulated in approximately 80% of HNSCC.^126^ Lymph node metastases with elevated MET levels can also be present.^127^ Overexpression of MET is recognized as a cause of EGFR inhibitor resistance, as it compensates for PI3K and MAPK inhibition in EGFR signaling.^123,128^ Anoikis resistance is enhanced in HNSCC as a consequence of HGF amplification, which is essential for developing nodal metastasis.^127^ KRT16 overexpression is associated with metastasis, increased mortality rate, unfavorable pathological differentiation, and advanced stages in OSCC patients. c-Met was also discovered to correlate with KRT16 through β5-integrin.^129^

#### Wnt/β-catenin pathway

Various components of the Wnt/β-catenin signaling pathway, including Wnt ligands, Wnt inhibitors, membrane receptors, and intracellular mediators, are regularly impaired by genetic alterations in malignant tumors, such as OSCC.^130–133^ The Wnt/β-catenin signaling pathway determines cell fate and proliferation in OSCC, whereas aberrant Wnt/β-catenin signaling promotes oncogenesis, typically via various mechanisms related to abnormal β-catenin stimulation.^134^ For example, KMT2D, one of the most frequently mutated genes in OSCC cells, collaborates with MEF2A to boost the transcription activity of β-catenin.^135^ In addition, the SNHG17/miR-384/ELF1 axis stimulates the Wnt/β-catenin pathway by upregulating CTNNB1 expression to drive the proliferation and metastasis of OSCC cells.^136^ DEP domain containing 1 (DEPDC1) is also essential for OSCC progression, which drives OSCC metastasis and aerobic glycolysis through the WNT/β-catenin pathway.^137^ Mutations that inactivate NOTCH1 and FAT1 diminish their capability to suppress the expression of β-catenin.^138^ In addition to mutations in Wnt/β-catenin signaling components, OSCC exhibits an overexpression of Wnt ligands.^139^ A high level of Wnt-7b in OSCC activates Wnt/β-catenin and facilitates cancer cell invasion and proliferation.^140^ Moreover, Wnt7a enhances the expression of MMP-9 to facilitate OSCC progression.^141^ Overall, Wnt/β-catenin signaling has a vital role to play in the formation of oral malignancies.

#### RAS/RAF/MAPK pathway

Only 4% of HNSCC cases exhibit mutations of mitogen-activated protein kinase (MAPK) signaling, which modulates cell proliferation, death, differentiation, angiogenesis, and dissemination.^142–144^ It comprises four sub-pathways, namely extracellular signal-regulated kinase (ERK1/2), c-Jun N-terminal kinase (JNK), p38, and ERK5 sub-pathways.^144^ In oral cancer, the ERK1/2 pathway has generated significant interest due to the fact that ERK1/2 is activated mechanistically by binding the growth factor EGF. Erl1/2 separates from RAS-RAF-MEK-ERK1/2 and induces the phosphorylation of OSCC-causing transcription factors, such as c-Myc, ETS-1, AP-1, NF-κB, and others.^144^ Meanwhile, OSCC cell growth is influenced by various proteins serving as targets, including SH3 domain-containing kinase binding protein 1 (SH3KBP1),^145^ annexin A10,^146^ fibroblast activation protein,^147^ EGFR,^148^ parathyroid hormone-related protein,^149^ angiopoietin-like 3 (ANGPTL3),^150^ quaking 5,^151^ and 70-kDa ribosomal S6 kinase.^152^

### Aberrant inactivation of suppressor signaling

TP53/RB, p16/Cyclin D1/Rb, and NOTCH are examples of suppressor signaling pathways. During the malignant transformation of OPMDs to OSCC, they become abnormally inactivated and downregulated.

#### TP53/RB pathway

Approximately 80% of HPV^-^ HNSCC have muted tumor protein p53 (TP53), resulting in gene dysfunction.^138,153,154^ Exon 4 or intron 6 is the location of TP53 mutations that occur early in the progression of HNSCC, especially OSCC.^155^ Consistently, p53 mutation is commonly observed in HPV^-^ OSCC, as the HPV E6 oncoprotein degrades p53.^156^ In both subtypes, mutations in p53 are correlated with a lower overall survival rate, treatment resistance, and an increased risk of relapse.^138^ In early OSCC, *TP53* expression is also associated with tumor stage and grade, as well as surgical margin dysplasia. It is not clear, however, whether TP53 expression and lymph node metastasis are related.^157,158^ p53 modulates cell death, apoptosis, and differentiation in OSCC cells by interacting with a complicated network of proteins.^138,159^ APR-246 targets GSTP1 to reactivate p53 and induce cell dealth.^160^ Co-expression of platelet-derived growth factor receptor A (PDGFRα) and p53 stimulates cell growth in poorly differentiated OSCC.^161^ Accordingly, TP53 and p53 collaborate to enhance OSCC invasion.^162^

Similar to mutations in the TP53 pathway, retinoblastoma (RB) pathway mutations are early manifestations of HNSCC. Both p53 and RB pathway mutations contribute to the unrestricted replication of HNSCC cells.^100^ In HPV^+^ neoplasms, the degradation of pRb by E7 contributes to the secretion of E2F and unregulated HNSCC cell proliferation.^163^ In persistent HPV infection, E2-regulated expression of E6 and E7 is responsible for p53 degradation and Rb functional suppression.^150^ When pRb is dysregulated, oral epithelial dysplasia has an increased likelihood of transforming into malignant carcinomas.^164^

#### p16/Cyclin D1/Rb pathway

In most HNSCC, the tumor suppressor p16 is inactivated, resulting in aberrant cell cycle control and cell proliferation, a deficiency in cell senescence, and ultimately dysplasia.^165^ Similar to HNSCC, OSCC frequently has a low level of p16.^166,167^ OSCC patients with inactive p16 tend to have a lower survival rate than those with normal or augmented p16 levels.^168^

Cyclin D1 (CCND1) amplification occurs in 25–43% of OSCC cases.^169–171^ In the early stages of OSCC, CCND1 is upregulated and contributes to the proliferation of OSCC cells.^172^ Particularly, CCND1 is more likely cytoplasmically expressed in advanced OSCC with deleterious differentiation, increased mitosis, and invasive cell morphology.^173^ Elevated expression of CCND1 is also related to reduced overall survival and poor prognosis among patients with OSCC.^174,175^ CCND1 deficiency inhibits the cyclin-dependent kinases CDK4 and CDK6, which are responsible for the cell cycle progression by dephosphorylating and inactivating pRb and then hindering G1 to S transition.^176^

#### NOTCH pathway

Notch signaling shows diverse effects on different cell types.^177–181^ Therefore, the suppressive or oncogenic functions of Notch in tumorigenesis have a contextual basis. According to a 2015 TCGA analysis, NOTCH1-3 is inactivated in 17% HPV^+^ and 26% HPV^-^ HNSCC.^138^ The majority of these aberrations are found in NOTCH1, including nonsense mutations causing truncated proteins, missense mutations within functional regions, and frameshift deletions and insertions. On the basis of mutational features, such as the absence of mutational hotspots and the presence of nonsense mutations, it is hypothesized that NOTCH1 acts as a tumor suppressor in HNSCC.^182^ Notch signaling has been demonstrated as a tumor suppressor in epithelial SCC malignancies (lung, bladder, and esophageal tumors) and several in vivo models.^159^

Nevertheless, in vitro assays with HNSCC cell lines have shown a requirement for an increase in Notch signaling activity to maintain malignant behavior.^183^ Recent research has shown that 43% of OSCC cases from a Chinese population are associated with activating mutations in NOTCH1, including novel mutations in heterodimerization and abrupted domains likely to obtain function.^184^ Furthermore, the mutation of NOTCH1 can result in a poor prognosis and lymph node metastasis in OSCC.^185^ NOTCH1 is responsible for sustaining the characteristics of cancer stem cells (CSCs), which are essential for cancer relapse and migration, via Wnt signaling; and 32% of HNSCC showed overexpression of downstream Notch effectors (measured by methylation, DNA copy number, and expression of 47 genes involved in Notch signaling).^186^

Overall, it remains unclear whether NOTCH mutations in HNSCC are typically activating or inactivating.^184,187^ Various mutations may be present in distinct subtypes of HNSCC.^177^ Since in vitro assays may not accurately reflect the disease progression in patients, it is imperative to investigate the functional role of Notch signaling in OSCC using robust in vivo models. Clinical trials of inhibitors or stimulators of the Notch pathway must be carefully considered.^188,189^

## Epigenetic modifications promote the development of OSCC

Epigenetic regulation refers to heritable and stable alterations in gene expression that do not modify the DNA sequence and are responsible for the formation and progression of OSCC neoplasms by regulating gene expression.^190–194^ Epigenetic modifications comprise DNA methylation, histone covalent modification, chromatin remodeling, and the impact of ncRNAs on gene expression (Table 2).^195^

**Table 2:** Epigenetic modifications in OSCC

Epigenetic modifications	Targets	Exhibitions	Outcomes
DNA methylation	▪ p16 ▪ MGMT ▪ MLH1 ▪ p15^INK4B^ ▪ E-cadherin ▪ PTEN ▪ APC ▪ P14^ARF^ ▪ P16^INK4A^ ▪ miR-137 ▪ miR193a	▪ Hypermethylation	▪ Oncogenic
	▪ AIM2 ▪ CEACAM1 ▪ LINE-1 ▪ PI3 ▪ PTHLH	▪ Hypomethylation	▪ Oncogenic
Histone acetylation	▪ H3K9ac ▪ H3K4ac ▪ miR-154-5p	▪ Downregulation	▪ Oncogenic
	▪ H3K27me3 ▪ HDAC6 ▪ HDAC8 ▪ HDAC1 ▪ HDAC2	▪ Upregulation	▪ Oncogenic
Chromatin modification	▪ SATB1 ▪ ZSCAN4 ▪ CSC factors ▪ RSF-1	▪ Upregulation	▪ Oncogenic
ncRNAs	▪ miR-1246 ▪ miR-31 ▪ miR-214 ▪ miR-23a ▪ miR-372	▪ Upregulation	▪ Oncogenic
	▪ miR-181a ▪ miR-17-92 cluster ▪ miR-329 ▪ miR-410 ▪ miR-211 ▪ TCF12 ▪ miR-214 ▪ miR-23a ▪ miR-372	▪ Upregulation	▪ Tumor suppression
	▪ lncRNA HOXA11-AS ▪ ZBTB7A ▪ miR-98-5p ▪ miR-214-3p ▪ circITCH	▪ Downregulation	▪ Oncogenic

### DNA methylation

The development and prognosis of OSCC are affected by DNA methylation abnormalities.^196^ Both hypomethylation and hypermethylation increase the prevalence of oral malignancies. In addition to the correlation between smoking and global hypomethylation,^197^ alcohol consumption is linked to evaluated levels of CpG hypermethylation in genes associated with oral cancers.^198^ Several OSCC samples have been found to manifest both hypermethylation and hypomethylation, leading to abnormal expression of genes, the majority of which are implicated in the tumorigenic process of OSCC by stimulating the Wnt and MAPK pathways.^199,200^

Particularly, DNA methylation silences suppressor genes in OSCC.^201,202^ CpG island hypermethylation inhibits over 40 tumor suppressor genes, which regulate the cell cycle, programmed cell death, Wnt pathway, cell-to-cell adhesion, and DNA repair in OSCC.^203^ The specific pattern of gene methylation leads to hypermethylated promoters in 22%-76% of OSCC patients.^193,204,205^ In contrast, the p16 promoter region was found to be methylated in only 5.4% of normal mucosa samples, indicating the epigenetic silencing of p16 in the development of OSCC.^193^ Moreover, DNA is methylated throughout the entire genome in 28-58% of premalignant oral tissues of tobacco users, with methylation levels increasing as the cancer progressed.^193^ Continued smoking increases DNA methyltransferase activity, enhancing methylation of the p16 promoter.^206,207^

OSCC also demonstrates hypermethylation in the promoter regions of many other genes, including O-6-methylguanine-DNA methyltransferase (MGMT), mutL homolog 1 (MLH1), and p15^INK4B^. MGMT functions in DNA repair by eliminating guanine DNA adducts, and maintaining the integrity of the genome.^191^ Therefore, increased MGMT levels render normal cells more resistant to carcinogens and spontaneous mutations.^208^ Silencing MGMT is associated with a poor prognosis in the early stages of OSCC development.^198,200^ Furthermore, the hypermethylation of MLH1 is essential for DNA mismatch repair and prevents the accumulation of DNA mutations, which are linked to the initiation of OSCC.^209^ Due to its significance, methylation patterns of MLH1 have been extensively investigated.^200^

In addition, p15^INK4B^ significantly contributes to tumor suppression. It inhibits cell proliferation and, consequently, cell cycle progression at the G1 stage, which is induced by stimulation of extracellular TGF-β and IFN-α.^200^ Hypermethylation of p15^INK4B^ may render cells less sensitive to these external stimuli, thereby influencing the progression of OSCC. Normal tissues lack methylation of p15^INK4B^; therefore, its abnormal methylation can serve as an indicator for OSCC.^210^ Additionally, hypermethylated E-cadherin,^211^ phosphatase and tensin homolog (PTEN),^212^ adenomatous polyposis coli (APC),^213^ p14^ARF^,^214^ p16^INK4A^,^215^ miR-137,^216^ and miR-193a^217^ prevent oral cells from promoting OSCC.^218^

Hypermethylation can also lead to the suppression of genes involved in the progression and metastasis of OSCC. For example, DNA methyltransferase (DNMT) levels have been linked to OSCC progression, growth, poor prognosis, and a higher risk of metastasis.^219^ DNMT3a immunoreactivity increased significantly in OSCC tissues compared to normal tissues.^220^ Despite some reports of normal DNMT1 expression in OSCC, the preponderance of studies have demonstrated that OSCC development is associated with DNMT overexpression.^221^ In general, DNMT1 regulates the prognosis of OSCC patients in a manner that decreases their overall survival.^222^

In alcoholic beverages, ethanol and acetaldehyde cause DNA hypomethylation.^223^ Global hypomethylation may facilitate tumorigenesis by diminishing the methylation of CpG dinucleotides across the entire genome.^194^ Moreover, global hypomethylation may promote the progression of cancer by demethylating previously methylated promoter regions of numerous oncogenes, thereby altering their expression.^200^ The presence of these characteristics is linked to the progression of malignant tumors, as they can increase the instability of the genome.^191^ AIM2,^224^ CEACAM1,^225^ LINE-1,^226^ PI3,^227^ and PTHLH^228^ are found as hypomethylated genes that contribute to OSCC.^218^

### Histone and chromatin modification

There are two distinct forms of chromatin: heterochromatin, which is highly compressed and transcriptionally silent, and euchromatin, which is less dense and transcriptionally active. The organization of chromatin and the expression of genes depend heavily on histone modifications, particularly histone acetylation. It is therefore not surprising that abnormalities in histone acetylation correlate with the progression of oral malignancies.^229^ Recent studies have identified reduced histone H3K9ac as an indicator of chemoresistance related to the NFκB pathway and CSCs recruitment^230–232^; it is also associated with enhanced cell proliferation and disruption of EMT in oral tumorigenesis.^233^ In light of these findings, H3K9ac assists in HNSCC development. Furthermore, low H3K4ac and high H3K27me3 levels are related to disease-free survival (DFS) and cancer-specific survival (CSS) and influence the progression of OSCC.^234^

In contrast to other histone modifications (such as methylation and phosphorylation), which have been extensively investigated in various cancer types, studies on the role of acetylation in the formation of OSCC are relatively limited. The acetylation of histones is governed by the equilibrium between histone acetyltransferases (HATs) and histone deacetylases (HDACs). Multiple HDACs are correlated with differentiation, cell cycle-associated genes, programmed cell death, angiogenesis, and dissemination of cancer cells, which have been observed to be altered in OSCC.^235,236^ OSCC exhibits overexpression of HDAC1,^237^ which contributes to OSCC growth and progression by regulating miR-154-5p/PCNA signaling.^238^ HDAC2 mRNA and protein levels are higher in OSCC and premalignant lesions than in controls. Meanwhile, the protein level of HDAC2 is related to histological differentiation and the stage of tumor-node-metastasis (TNM) in OSCC patients, emphasizing its significance in the progression of premalignant to malignant carcinomas.^239^

Chromatin remodelers are crucial molecules in charge of modulating nucleosome positioning and chromatin accessibility in response to DNA-driven stimulation of biological processes in OSCC.^240^ For instance, special AT-rich sequence binding protein 1 (SATB1) is a genome-organizing protein that modifies chromatin structure and directs chromatin remodeling enzymes to specific chromatin regions to regulate gene expression. High SATB1 expression is related to HNSCC metastasis, poor prognosis, and decreased survival rate.^241^ The zinc finger and SCAN domain containing 4 (ZSCAN4) is another example of a protein that can alter the epigenetic profile and chromatin state in tumors.^242^ ZSCAN4 induces the functional hyperacetylation of histone 3 at the OCT3/4 and NANOG promoters, thereby upregulating CSC factors.^77^ In contrast, ZSCAN4 exhaustion contributes to the downregulation of CSC markers, the diminished capacity to develop tumorspheres, and the restriction of tumor growth.^243^ Therefore, ZSCAN4 is essential for maintaining the CSC phenotype and tumor progression in HNSCC.^77^ Furthermore, the chromatin remodeler RSF-1, a member of the ISWI family,^244^ is upregulated in OSCC and linked to enhanced invasion, lymph node metastasis, and advanced stages of carcinomas. RSF-1 exhibits the ability to increase the resistance of OSCC cells to both radiotherapy and chemotherapy.^245^

### Non-coding RNAs

Similar to proteins, microRNA (miRNA) dysfunction can arise from abnormalities in miRNA expression caused by genetic mutations, epigenetic modifications, or processing deficiencies. MicroRNAs are a class of ncRNAs that have been intensively investigated in HNSCC. Due to genetic and epigenetic alterations, a number of miRNAs have been linked to OSCC.^246^ miR-26,^247^ -137,^248^ and -203^249^ are repressed by CpG hypermethylation in OSCC. Notably, miR-26a binds to the DNMT3B enzyme, which stimulates cell proliferation, indicating an intimate relationship between epigenetic modifications and the progression of oral malignancies.^250^

miRNAs are capable of functioning as either tumor oncogenes or suppressors to modulate cell growth.^251^ For instance, miR-1246 functions as an oncogene and is abundant in exosomes derived from the oral carcinoma cell line HOC313-LM, which is highly metastatic.^252^ After the transfer of miR-1246 via exosomes, inadequately metastatic cells displayed enhanced cell motility and invasion capability. miR-1246 facilitates cell mobility by directly binding to DENN/MADD Domain Containing 2D (DENND2D) in OSCC.^253^ In addition, patients with oral malignancies had increased levels of miR-31 in their saliva throughout the entire process of the disease.^254^ Following oral tumor excision, the level of salivary miR-31 decreased significantly, indicating that the majority of the raised level of salivary miR-31 originated from tumor tissues.^255^

OSCC is frequently associated with the downregulation of numerous tumor-suppressing miRNAs. For example, ectopic miR-181a expression inhibits the proliferation and anchorage-independent growth of OSCC.^256^ miR-181a significantly inhibits OSCC formation in three-dimensional organotypic raft cultures.^257^ Mechanistically, miR-181a reduced K-ras protein levels and luciferase activity in receptor vectors containing the 3’-untranslated region of the K-ras gene. By inhibiting the oncogene K-ras, miR-181a could suppress OSCC.^257^ Similar results are observed when the miR-17-92 cluster, comprising miR-17, miR-19b, miR-20a, and miR-92a, is induced to overexpress in OSCC cell lines.^258^ miR-17/20a within the miR-17-92 cluster modulates OSCC migration predominantly and is inversely associated with TNM stage and lymphatic metastasis in clinical investigations; it is known to suppress tumor migration in OSCC.^258^ Other examples include miR-329, miR-410, and miR-211. Increased levels of miR-329 and miR-410 inhibit the proliferation and dissemination of OSCC cells. Specifically, miR-329 and miR-410 could bind to Wnt-7b and then attenuate the Wnt-β-catenin pathway in OSCC.^140^ miR-211 is upregulated in OSCC cells by arecoline and 4-nitroquinoline 1-oxide (4NQO), and it directly inhibits OSCC cell growth by targeting transcription factor 12 (TCF12). However, miR-211 levels are drastically reduced in tumor tissues, resulting in an enhanced oncogenicity for OSCC.^259^

The progression of OSCC cells toward a drug-resistant state appears to be mediated by multifunctional ncRNAs, including miRNA, lncRNA, and circular RNA (circRNA).^260^ Over 10% of all identified miRNAs correlate with chemotherapy resistance in cancer cells, particularly those implicated in OSCC cell chemoresistance.^261–263^ In the presence of cisplatin (CDDP) resistance, miR-214 and miR-23a levels were promoted, as determined by microarray analysis.^264–266^ miR-372 is overexpressed in OSCC and inhibits zinc finger and BTB domain-containing 7A protein (ZBTB7A), promoting tumorigenesis and CDDP resistance in OSCC cells.^267,268^ OSCC cells and tissues overexpress the lncRNA HOXA11-AS relative to adjacent normal tissues and oral keratinocytes.^269^ lncRNA HOXA11-AS consumes miR-98-5p, which is capable of inhibiting OSCC cell proliferation,^270^ and suppresses miR-214-3p expression, which may lead to the establishment of drug resistance in OSCC.^271^ In addition, when circITCH is upregulated, chemotherapy agents are more effective against drug-resistant myeloma cells. However, OSCC tissues and cells express less tumor-suppressing circITCH than adjacent normal tissues and human oral keratinocytes.^272^

In addition to the aforementioned characteristics, OSCC exhibits phenotypic plasticity. EMT is an epigenetically regulated process that induces plasticity in OSCC and leads to the transition of cancerous cells into distinct phenotypic forms with increased motility and survival. Notably, oral CSC plasticity interacts with EMT to enhance treatment resistance in OSCC.^273–276^

## Dysregulated tumor microenvironment in OSCC

OSCC occurrence and progression are influenced by a dysregulated tumor microenvironment. Specifically, a suppressed immune system, stromal alteration, hypoxia, and an unbalanced oral microbiome all contribute to the development and metastasis of OSCC, so their underlying mechanisms present therapeutic opportunities.

### Immune suppression

**Fig. 3:**
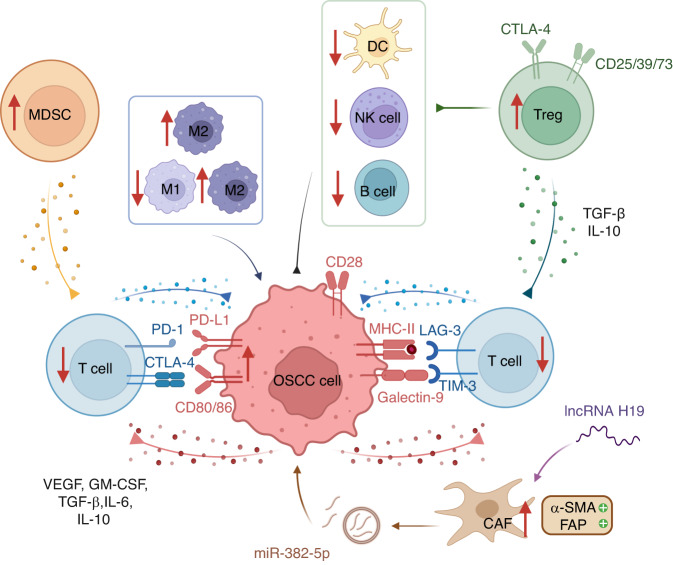
Immunosuppressive TME in OSCC. The tumor microenvironment (TME) contains various immunomodulatory cells, including cancer-associated fibroblasts (CAFs), regulatory T cells (Tregs), tumor-associated macrophages (TAMs), and myeloid-derived suppressor cells (MDSCs). The interaction between programmed death 1 (PD-1) and programmed death ligand-1 (PD-L1) leads to T-cell suppression and adaptive immunity tolerance, whereas cytotoxic T-lymphocyte-associated antigen 4 (CTLA-4) competes with CD28 by interacting with CD80/86 on OSCC cells. Other immune checkpoints also play a role, including LAG-3 and TIM-3. OSCC cells produce immune suppressive factors such as vascular endothelial growth factor (VEGF), granulocyte-macrophage colony-stimulating factor (GM-CSF), transforming growth factor (TGF)-β, interleukin (IL)-6, and IL-10, which block effector cells. Tregs generate TGF-β and IL-10 to diminish the functions of T cells. CAFs express α-smooth muscle actin (α-SMA) and fibroblast activation protein (FAP) and promote tumor growth by overexpressing miR-385-5p in their exosomes. In the TME, there is a greater proportion of M2 macrophages to M1 macrophages, with M2 TAMs possessing carcinogenic properties. OSCC oral squamous cell carcinoma

Patients with suppressed immune states, such as HIV^+^ patients and organ transplant recipients, are more likely to develop oral malignancies,^277–279^ suggesting that immune response plays a crucial role in OSCC development (Fig. 3).^92,280–283^

OSCC evades the immune surveillance of their hosts by employing a variety of molecular-level strategies. First, a high mutational burden in OSCC caused by smoking and alcohol abuse-induced DNA damage facilitates immune evasion.^86^ Particularly, OSCC is linked to mutations in human leukocyte antigen (HLA) and antigen processing machinery (APM) that are critical for evading immune surveillance.^284,285^ Second, immune checkpoints contribute to immune evasion of OSCC. An estimated 83% of OSCC expresses programmed death ligand-1 (PD-L1), which interacts with programmed death 1 (PD-1) on T cells and induces T-cell suppression and tolerance to adaptive immunity.^286^ The expression of cytotoxic T-lymphocyte-associated antigen 4 (CTLA-4) is elevated in OSCC for evading immune surveillance. CTLA-4 interacts with CD80/86 on antigen-presenting cells (APCs) to compete against its stimulatory counterpart CD28 to block the differentiation of naïve T cells.^287^ Additionally, significant immune checkpoints such as lymphocyte activation gene-3 (LAG-3),^288^ T-cell immunoglobulin and mucin-containing protein-3 (TIM-3),^289^ and B7 Homolog 3 (B7-H3)^290^ are overexpressed in OSCC. Third, cancer cells can secrete cytokines that suppress adaptive immunity and promote tumor growth. Particularly, OSCC cells generate immunosuppressive inflammatory cytokines, such as VEGF, granulocyte-macrophage colony-stimulating factor (GM-CSF), TGF-β, IL-6, and IL-10, which affect T cells.^291^ Moreover, a significant reduction of immune-activating cytokines, like IL-2, inhibits the stimulation of the innate and adaptive immune response against OSCC.^292^

Microenvironment regulators such as hypoxia, abnormal vasculature and lymphatics, and high interstitial pressure, may also manipulate the immune response to OSCC by influencing the secretion of cytokines, the trafficking of immune cells, and the function of the immune system. In addition, patients with uncommon inherited diseases such as FA and DC exhibit immune deficiencies that promote OSCC progression.^293,294^

FA is an autosomal recessive genetic disorder. It manifests as aplastic anemia, progressive pancytopenia, congenital anomalies, and an elevated incidence of developing malignancies. In a study conducted in Brazil, 121 cases of oral malignancies in FA patients were identified.^293^ Hematopoietic stem cell transplant (HSCT), the only current treatment option for the hematological complications of FA patients, is linked to a 500-fold increased risk of head and neck malignancies and a risk factor for a more rapid progression of oral malignancy in comparison to non-transplanted patients.^295^ DC is a rare genetic disorder characterized by premature telomere shortening that leads to bone marrow failure. DC-associated mucocutaneous illness symptoms include reticulated pigmentation of the skin, nail dysplasia, and oral leukoplakia. Multiple malignancies may develop in patients with DC, such as a transition from leukoplakia to HNSCC.^294,296^

Taken together, the above-mentioned immunosuppressive tumor microenvironment enables OSCC to evade immune recognition and elimination, thereby presenting therapeutic opportunities.

### Stromal alteration

Due to stromal alteration, HPV^-^ and HPV^+^ OSCC can escape the cytotoxic mechanisms despite overexpressing CD8^+^ cytotoxic T cells and activated NK cells. This is influenced by various stromal cells, including CAFs, tumor-associated macrophages (TAMs), myeloid-derived suppressor cells (MDSCs), and regulatory T cells (Tregs) (Fig. 3).^297^ These stromal cells can also secrete cytokines and immune checkpoint inhibitors, which act together to constitute an immunosuppressive microenvironment and allow the growth of neoplasms.^291^

Active CAFs express α-smooth muscle actin (α-SMA) and fibroblast activation protein (FAP), which promote OSCC metastasis.^298^ CAFs boost OSCC development via miR-382-5p overexpression in their exosomes and lncRNA-regulated RUNX2/GDF10 signaling. Meanwhile, lncRNA H19 enhances glycolysis of CAFs in the oral cavity through the miR-382-5p/PFKFB3 (6-phosphofructo-2-kinase/fructose-2,6 biphosphatase 3) axis.^299^ In addition, senescent CAFs facilitate the rapid progression of oral malignancies in genetically unstable OSCC by promoting keratinocyte migration, inhibiting epithelial adhesions, and releasing active matrix metalloproteinases-2 (MMP-2).^300–303^ CXCL1^304^ and peroxiredoxin1 (PRDX1)^305^ drive the development of OSCC through the induction of cell senescence.

TAMs consist of M1 TAMs with antitumor properties and M2 TAMs with tumorigenic properties.^306–308^ A higher ratio of M2 to M1 TAMs is commonly observed in the peritumoral microenvironment surrounding OSCC, promoting carcinogenesis.^309,310^ MDSCs are immature myeloid-derived cells which can suppress T cells.^311–315^

Patients with OSCC have an elevated quantity of Tregs in their peripheral circulation, lymph nodes, and neoplasms.^277–279,316^ Tregs are able to suppress various stromal cells, including CD4^+^ and CD8^+^ T cells, B cells, dendritic cells (DCs), and natural killer cells (NKs),^317,318^ due to the expression of CTLA-4, CD73, CD39, and CD25, and the production of TGF-β, IL-10, and perforin/granzyme B.^319–322^ It has been demonstrated that mice with OSCC have elevated levels of immunosuppressive CD11b^+^Gr-1^+^ cells in the peripheral circulation, spleen, and tumors.^323^ Tumor CD11b^+^Gr-1^+^ cells express more PD-L1 than cells from other tissues, disrupt T-cell proliferation in vitro, and ultimately suppress immunity.^323^ On the other hand, researchers hypothesize that elevated T-helper 2 cells in the immune system may shield individuals with allergies or asthma from tumor development;^324^ however, additional investigation is required to verify if this is true.

### Hypoxia

**Fig. 4:**
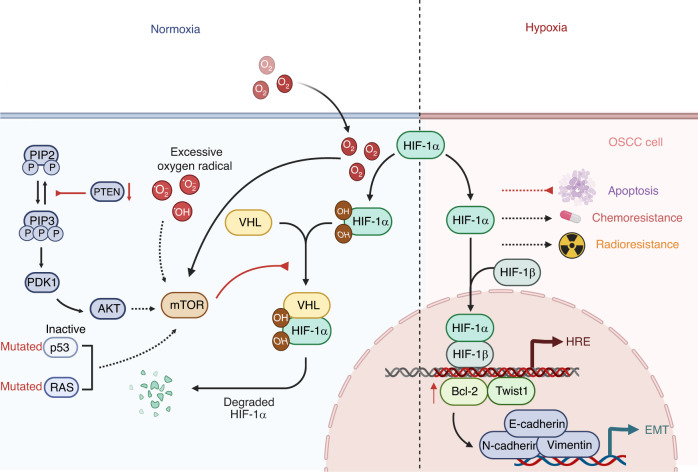
Hypoxia in OSCC. Under normoxic conditions, VHL degrades the HIFα subunits. In the condition of hypoxia, HIFα becomes stable and binds to HIFβ within the nucleus, adhering to hypoxia HREs to enable tumor adaptation. Hypoxia also promotes Bcl-2/Twist1 interaction by enhancing Bcl-2 attachment to Twist. Particularly, the mTOR pathway has been demonstrated to increase the level of HIFα in tumor regions that do not experience significant hypoxia. Several oncogenic mechanisms, such as inactive p53 mutations, RAS mutations, excessive oxygen radical accumulation, suppression of PTEN, and the infective HIF-1α degradation by VHL mutation, have been identified as contributing to this development. In addition, HIF-1α stimulates Twist1 to transactivate EMT-related genes, including Vimentin, N-cadherin, and E-cadherin, in order to drive EMT. Moreover, HIF-1α blocks apoptosis and confers higher resistance to chemotherapy and radiotherapy on OSCC. EMT epithelial–mesenchyme transition, HERs hypoxia response elements, HIF hypoxia-inducible factors, OSCC oral squamous cell carcinoma, PTEN phosphatase and tensin homolog, VHL Von Hippel-Lindau

OSCC is a locally aggressive tumor with an elevated hypoxia level, resulting in dissemination, relapse, and poor therapeutic response (Fig. 4).^285,325–328^ Of note, hypoxia is induced by hypoxia-inducible factors (HIF) in OSCC.^329,330^ HIFs 1-3 are the principal hypoxia response mediators. Under normoxic conditions, the E3 ubiquitin ligase Von Hippel-Lindau (VHL) protein degrades the HIFα subunits.^331,332^ In the presence of hypoxia, HIFα becomes stable and binds to HIFβ in the nucleus, adhering to hypoxia response elements (HREs) to facilitate tumor adaptation.^333^ HREs are present in genes involved in metabolism, extracellular matrix remodeling, angiogenesis, immune modulation, and inflammation.^334–337^ In addition, hypoxia promotes Bcl-2/Twist1 interaction by enhancing Bcl-2 attachment to Twist1, which is related to the poor prognosis of OSCC patients.^325,338^

Several examples demonstrate that HIFs can stabilize under normoxic conditions, suggesting that hypoxia is not clearly defined. In particular, the mTOR pathway raises the level of HIF-1α in tumor regions that do not experience significant hypoxia. A variety of oncogenic mechanisms, such as inactive p53 mutations, RAS mutations, excessive oxygen radical accumulation, suppression of PTEN, and infective HIF-1α degradation due to VHL mutations, have been identified as contributing to this development. HIF signaling is synergized with activating mutations in p53.^335,339,340^

Hypoxia and EMT have a correlation with OSCC metastasis and invasion.^325^ Hypoxia-induced decreases in E-cadherin mRNA levels boost the migration capability of OSCC cells.^341^ HIF-1α drives EMT by stimulating Twist1 to transactivate EMT-related genes, such as Vimentin, N-cadherin, and E-cadherin.^342^ Moreover, HIF-1α blocks apoptosis and imparts increased chemoresistance and radio-resistance in OSCC, thereby contributing to the aggressiveness of the disease.^343^

BQ chewers exhibit endogenous nitrosation, which generates potentially carcinogenic nitrosamines, including 3-methylnitrosopropionitrile.^344^ As a result of the auto-oxidation of polyphenols found in areca nuts, reactive oxygen species (ROS) are present in the mouths of patients who consume BQ, which is exacerbated by the alkaline pH of slaked lime^345^ and can stimulate hypoxic adaptation in OSCC cells.^346^

Taken together, as HPV^-^ HNSCC are susceptible to p53 mutations and are hypoxic, the synergy between p53 mutants and HIF-1 signaling may provide a valuable avenue for future research. Hypoxia tumors are a subtype of OSCC characterized by a poor prognosis and resistance to treatment.^287^

### Oral microbiome

**Fig. 5:**
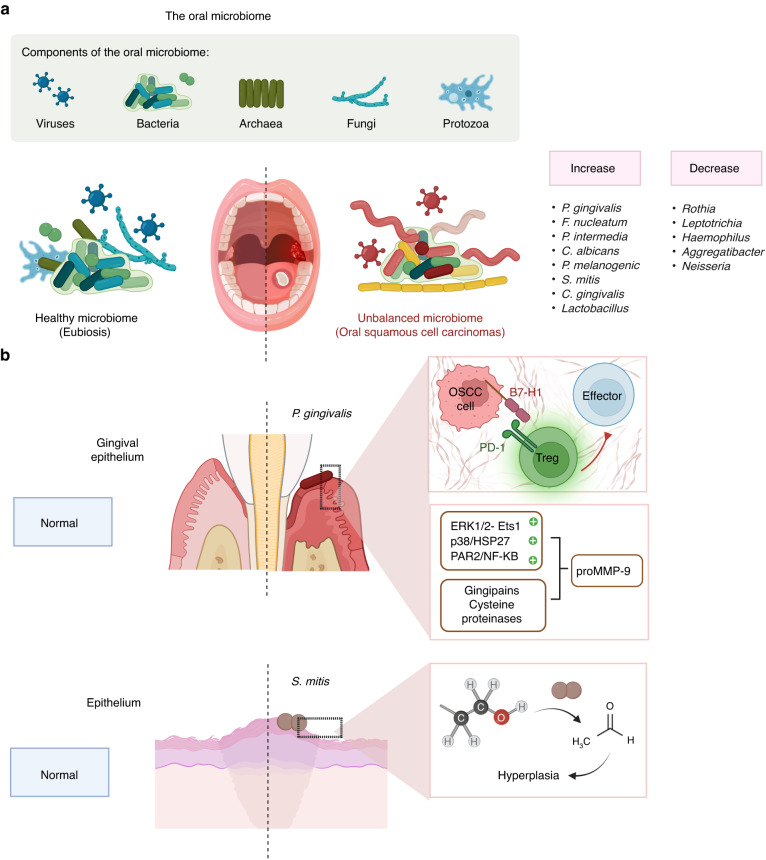
Dysbiosis in OSCC. **a** A correlation exists between dysbiosis and the occurrence of various cancers. OSCC could be caused by *Lactobacillus, Porphyomonas gingivalis, Fusobacterium nucleatum*, and *Prevotella intermedia*. OSCC patients have elevated levels of *Prevotella melanogenic, Streptococcus mitis*, and *Capnocytophaga gingivalis* in their saliva, while *Rothia, Leptotrichia, Haemophilus, Aggregatibacter*, and *Neisseria* are diminished. **b** *P. gingivalis* stimulates the expression of B7-H1 receptors on OSCC cells. B7-H1 activates the development of Tregs, thereby inhibiting effector T cells. *S. mitis* has been demonstrated to trigger mutations and epithelial hyperplasia by generating acetaldehyde. OSCC oral squamous cell carcinoma, Tregs regulatory T cells

Independent of other risk factors, oral microbiome alterations may facilitate the progression of oral malignancies in 7–15% of cases.^347–351^ The microbiome is comprised of a complicated community of bacteria, fungi, protists, archaea, and viruses, all of which contribute to the maintenance of microbial diversity.^352–357^ Statistical evidence establishes a correlation between dysbiosis and the incidence of various cancers, thereby elevating the clinical significance of the oral microbiome (Fig. 5).^358^ In the diverse oral cavity environment, including mucosal surfaces and deep tissue crevices, there are distinct microbial species present in both healthy and malignant.^359^

Certain bacterial species have been linked to the development of oral carcinoma. Some periodontal bacteria, such as *Porphyomonas gingivalis*, *Fusobacterium nucleatum*, and *Prevotella intermedia*, may be responsible for OSCC.^360^ The presence of elevated levels of *Lactobacillus*,^361^ *Prevotella melanogenic, Streptococcus mitis*, and *Capnocytophaga gingivalis*^362^ in saliva can also be used to diagnose OSCC. OSCC patients have been found to lack *Rothia*,^363^ *Leptotrichia*,^364^ *Haemophilus*,^363^ *Aggregatibacter*,^365^ and *Neisseria*.^361^

*P. gingivalis* can stimulate OSCC cells to express the B7-H1 and B7-DC receptors.^366^ Expression of B7-H1 activates Tregs development, thereby inhibiting effector T cells. Consequently, the expression of B7-H1 by oral carcinomas may aid their ability to evade immunity.^366^ Infection with *P. gingivalis* induces ERK1/2- Ets1, p38/HSP27, and PAR2/NF-KB signaling to promote expression of promatrix metalloproteinase (proMMP-9).^360^ In this process, *P. gingivalis* produces gingipains, which are cysteine proteinases with a dual function, and then binds to the PAR2 receptor, resulting in the maturation of the proenzyme MMP-9 into its active form. The destruction of the basement membrane facilitates the invasion and metastasis of OSCC cells through blood vessels and lymphatic systems. Collectively, *P. gingivalis* contributes to the spread of OSCC.^367^

The oral microbiome is affected by OSCC risk factors such as smoking, alcohol abuse, and HPV infection.^368^ There is a correlation between exposure to these risk factors and a shift in diverse bacterial genera of bacteria. Also, oral microbes have been demonstrated to trigger mutations and epithelial hyperplasia by generating acetaldehyde, a cancer-causing derivative of ethanol.^369^ The ability of different bacterial strains to produce acetaldehyde varies considerably. For instance, *S. mitis* produces a substantial quantity of acetaldehyde and is an active alcohol dehydrogenase. OSCC has been found to contain elevated levels of *S. mitis*.^370^

In addition, OSCC has been observed in immunocompromised patients with chronic mucocutaneous candidiasis and rarely in patients with autoimmune polyendocrinopathy-candidiasis-ectodermal dystrophy.^371^ *Candida albicans* is more prevalent in the oral cavity of patients with OSCC or leukoplakia compared to those without oral pathology.^372,373^

## Therapeutic interventions and prognostic factors for OPMDs and OSCC

The goal of multidisciplinary therapeutic strategies for OPMDs is to impede the development of OSCC and reduce mortality and morbidity. However, due to the complexity and diversity of OPMDs, there has yet to be a consensus regarding the optimal treatment approach. Meanwhile, OSCC management is crucial for improving survival rates, especially considering the possibility of malignant transformation of OPMDs.

### Therapeutic interventions for OPMDs

OPMDs are treated differently depending on their classification. Although there are numerous methods for preventing and managing OL, there is no standard approach.^26^ According to the severity of the dysplasia, OL is typically treated surgically by excision of the lesions. Nevertheless, a study on leukoplakia surgery reveals that surgery decreases but does not completely eliminate the risk of developing leukoplakia. Also, randomized controlled trials have yet to be conducted to determine whether removing the OL reduces the likelihood of OSCC. According to a previous study, 20% of OL patients may recover if risk factors are eliminated and antifungal therapy is administered. Based on a retrospective study of 94 patients whose OL was surgically removed and 175 patients who did not undergo surgery, OSCC was found to occur in 12% and 4% of patients, respectively.^374^ Overall, surgery did not safeguard the inhibition of transformation.

As an alternative to OPMD surgery, chemoprevention has the potential to diminish the risk of developing cancer. Chemoprevention is effective when vitamin A, bleomycin, β-carotene, or retinoids are utilized.^375,376^ Despite their ability to induce therapeutic responses, frequent relapses were observed. Additionally, laser ablation, cryosurgery, and photodynamic therapy are effective treatments for OL. Recent studies have demonstrated statistically significant improvements in the clinical outcomes of OL following erbium: yttrium aluminum garnet laser compared to cold scalpel excision.^376^

In terms of OE, early treatment is recommended due to its high incidence of malignant transformation.^377^ Lesions exhibiting severe epithelial dysplasia on excisional or incisional biopsies should be completely removed under microscopic examination.^32^ Laser surgery is also a recommended treatment option for OE.^378^ OE can be effectively ablated with CO_2_ lasers which contributes to a low morbidity rate.^32^ Using natural agents as chemopreventative agents is an additional method of treating OE. Several natural agents, including curcumin, green tea extract, and Bowman-Birk inhibitor concentrate, are beneficial in the treatment of OE.^379,380^ Continuous monitoring is advised for lesions exhibiting minimal to moderate dysplasia. In addition, postoperative recurrences are more likely to occur when the initial lesion size is larger, with lesions more than 80 mm^2^ regarded as significant predictors of recurrence.^32^

Due to the complicated pathogenesis of OSF, it is difficult to ascertain the most effective treatment. OSF cannot be completely cured by a singular treatment modality. Therefore, the primary objective of OSF treatment is to improve the condition of the oral mucosa, alleviate the burning sensation, and facilitate mouth opening. There are a variety of therapies available, including medical, physical, and surgical treatments. In the initial stages of OSF, cessation of BQ chewing and therapeutic interventions may reduce or eliminate symptoms. In the intermediate or advanced phases of OSF, surgical treatments are necessary to alleviate pain in the mucosal epithelium of the mouth and to facilitate mouth opening.^40,381,382^

In general, OLP is treated with palliative care as opposed to curative care. Asymptomatic reticular lesions are unnecessary to treat, but continued observation is advised. The main objective of treatment is to diminish inflammation and relieve the symptoms.^383^ In most cases, topical steroids are required to treat symptomatic OLP. Corticosteroids are used intralesionally to combat erosive OLP. In cases that are more severe or resistant, systemic steroids are prescribed.^384,385^ Topical calcineurin inhibitors can be administered to patients who do not respond to corticosteroids.^384^ Furthermore, topical cyclosporine and systemic immunosuppressants have been used to treat OLP.^386^

### Therapeutic interventions for OSCC

**Fig. 6:**
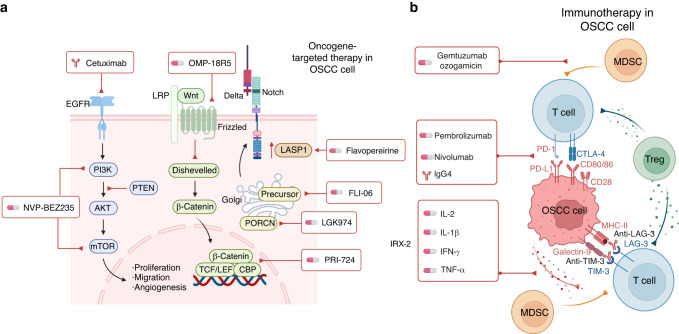
Treatments of OSCC. **a** Oncogene-targeted drugs facilitate OSCC chemotherapy. Cetuximab is an antibody that suppresses the EGFR pathway. NVP-BEZ235 is a PI3K/AKT/mTOR pathway inhibitor. Flavopereirine silences the JAK/STAT pathway and upregulates LASP1 expression. FLI-06 inactivates the Notch pathway. In the Wnt/β-catenin pathway, OMP-18R5 inhibits Fzd receptors; PRI-724 interrupts the interaction between β-catenin and CBP; and LGK974 targets PORCN. **b** Immunotherapy is an alternative treatment. Monoclonal antibodies pembrolizumab, nivolumab, and lgG4 have been authorized to target PD-1. LAG-3 and TIM-3 are blocked by their antibodies. IRX-2, comprising IL-2, IL-1β, IFN-γ, and TNF-α, is proven effective against inflammatory immune suppression cytokines in OSCC. Gemtuzmab ozogamicin promotes the differentiation of MDSCs into mature phenotypes, thereby reducing their immunosuppressive properties. EGFR epidermal growth factor receptors, PD-1 programmed death 1, OSCC oral squamous cell carcinoma

OSCC treatments include surgical intervention followed, if necessary, by postoperative radiation or chemotherapy. In most cases, surgery is the first-line treatment for oral carcinomas. In advanced cases, postoperative radiation, chemoradiation, oncogene-targeted therapy, and immunotherapy may be administered (Fig. 6).

Surgical procedures, such as open procedures, endoscopic procedures, and robotic surgery, are used to treat the majority of oral carcinomas. The purpose of surgical resection is to eliminate sufficient tumor tissue. Inadequate removal of tumor cells increases the likelihood of local and regional recurrences, thereby reducing long-term survival rates.^387,388^ In oral carcinoma surgery, a 1 cm margin of three-dimensional dissection is deemed appropriate.^389^ During primary tumor dissection, iodine solution staining is recommended to identify and delineate the dysplastic epithelium. However, a greater resection margin may enhance the risk of esthetic and functional complications in OSCC.^390,391^

Following the excision of the primary tumor, reconstructive surgery is typically required to restore oral cavity function and head and neck aspect. Postoperative oral disabilities can be reduced through routine surgical reconstruction. The choice of an appropriate method of reconstruction is affected by a variety of factors, such as the features of the primary defects, the medical history and general health of the patient, the skills of the surgeon, and the prognosis. In general, reconstructive procedures adhere to a “reconstructive ladder” consisting of a skin graft followed by a microvascular free flap. In the field of oral reconstruction, unrestricted tissue transfers are regarded as one of the most reliable and widely used techniques. There are various options for free flaps; however, no singular flap is capable of resolving the entire range of oral defects at present.^14,392^

Radiotherapy is typically administered postoperatively, as irradiated tissue cannot be removed surgically. Tissue fibrosis diminishes the effectiveness of regeneration. Radiotherapy at the primary site is determined by variables such as the primary tumor size, positive surgical margins, and the presence of perineural, lymphatic, and vascular invasion. Nevertheless, it is also common to treat the neck to prevent the possibility of metastasis and recurrence, particularly in lymph nodes with extracapsular dissemination. It is recommended to begin radiotherapy within six weeks after surgery. There are variations in the radiation doses, but an approximate cumulative dose of 60 Gy is typically provided.^393^ In addition, chemotherapy has recently become a popular adjunct treatment for locally advanced OSCC. Even though chemotherapy is not considered a curative treatment for oral carcinomas, it can be administered prior to surgery or in conjunction with irradiation before or after surgery. Adjuvant chemotherapy and radiotherapy are becoming standard remedies for advanced oral cancers. Other variants, such as daily low-dose and weekly intermediate-dose of CDDP, are also effective in improving survival rates.^394,395^ Typically, EGRF inhibitors are utilized to treat metastatic HNSCC.^14^

Furthermore, oncogene-targeted drugs can improve chemotherapy to OSCC.^396^ The antibody cetuximab has been shown to suppress the EGFR pathway and is approved for HNSCC treatment.^397^ NVP-BEZ235 inhibits the PI3K/AKT/mTOR pathway to sensitize OSCC cells to infrared radiation and diminish their resistance to radiotherapy.^398^ Flavopereirine silences JAK/STAT signaling and upregulates LASP1 to block the development of oral carcinomas.^399^ FLI-06 inactivates the Notch signaling pathway to disrupt the proliferation and self-renewal of oral malignancy cells.^400^ In the Wnt/β-catenin pathway, OMP-18R5 inhibits Fzd receptors,^401^ whereas PRI-724 interrupts the interaction between β-catenin and CBP.^402^ LGK974 targets PORCN, an acyltransferase vital for producing Wnt proteins in various carcinomas.^403^ Targeted drugs hold great potential for OSCC treatments in the clinic.

Immunotherapy is an alternative treatment. OSCC is considered to be an immunosuppressive disease. It has been suggested that a malfunctioning immune system participates in the development or recurrence of OSCC. Immunotherapy has shown promise in the handling of OSCC. For instance, anti-PD-1/PD-L1 agents promote an immune response against tumors by blocking the suppression signals of the immune checkpoints.^404^ Pembrolizumab, nivolumab, and lgG4 monoclonal antibodies that target PD-1 have been authorized for therapeutic interventions of metastatic HNSCC based on proven efficacy in clinical trials.^405,406^ Cytotoxic CD8^+^ T cells are recruited to suppress the growth of tumors in vivo by blocking LAG-3 with its antibodies.^407^ Also, anti-TIM-3 therapy appears to inhibit neoplasm growth in in vivo models.^408^ IRX-2, a multi-cytokine biologic preparation derived from homologous cells and comprised of IL-2, IL-1β, IFN-γ, and TNF-α, is under investigation and has been shown to be effective against inflammatory immune suppressive cytokines. To boost both adaptive and innate immune defenses against tumor cells present in the host, IRX-2 blocks tumor-induced apoptosis of T cells and facilitates their effector function in regional lymph nodes.^409^ In addition, anti-Treg receptor monoclonal antibodies are being developed to decrease the quantity of Tregs in the tumor microenvironment.^410^ Gemtuzumab ozogamicin facilitates MDSCs maturation, thereby reducing their immunosuppressive properties.^411^ An alternative strategy is to inhibit the recruitment of TAMs and MDSCs into the TME by blocking their chemotactic receptors.^412^

### Prognosis of OSCC

As mentioned above, multidisciplinary team care is essential to the effective treatment of OSCC. However, malignant tumors have a poor prognosis, with limited improvements in survival over many decades.^413–415^ After malignant transformation, it is crucial to evaluate the progression and development of OSCC in terms of angiogenesis, tumor budding, perineural invasion, staging, HPV status, and the presence of specific biomarkers. These prognostic factors help to assess the mortality rate of OSCC patients and guide treatment decisions (Table 3).^416–418^

**Table 3:** Prognostic factors for OSCC

Prognostic factors	Indicators	Exhibitions	Outcomes
Staging	Clinical stages: ▪ Stage I ▪ Stage II ▪ Stage III ▪ Stage IV	5-year survival rate: ▪ 79.8% ▪ 70.0% ▪ 57.6% ▪ 53.9%	▪ Poor prognosis
Biomarkers	▪ G3BP1 ▪ B7-H6 ▪ FAM3C	▪ Upregulation	▪ Poor prognosis
Angiogenesis	▪ MVD ▪ HMGA2 ▪ Id-1	▪ Upregulation	▪ Poor prognosis
HPV status	▪ HPV^+^ ▪ p16^+^	▪ Upregulation	▪ Favorable prognosis
Tumor budding	▪ Snail ▪ Twist	▪ Upregulation	▪ Poor prognosis
Perineural invasion	▪ miR-21/phosphatase ▪ Tensin homologs ▪ MMP-2 ▪ NGF ▪ Tyrosine kinase A	▪ Upregulation	▪ Poor prognosis

#### Clinicopathological factors

Several immunohistochemical staining protocols can be used on patient tissue samples to identify clinicopathological alterations such as angiogenesis, tumor budding, perineural invasion, and staging. Oral malignancies have the capability of inducing blood vessel formation, which is crucial to the dissemination of tumors.

Angiogenesis in tumors is generally evaluated by quantifying the blood vessels (microvessel density, MVD) present in regions of the tissue.^419^ In addition to immunohistochemical staining techniques for identifying vessels, angiogenesis can be investigated by other methods, such as the Chalkley method and flow cytometry.^420^ The presence of marked angiogenesis was related to an elevated possibility of nodal metastases and may indicate the requirement for intensified adjunctive treatment following surgery. Moreover, angiogenesis in OSCC is associated with the parameters of size (T) and lymph node involvement (N), a reliable indicator of tumor relapse.^11^ HMGA2 regulates OSCC angiogenesis-related genes and correlates with both distant and lymph node metastasis.^421^ Patients with high HMGA2 expression have a worse 5-year survival rate. HMGA2-high samples exhibit more CD34-stained blood vessels and higher expression of VEGF-A, VEGF-C, and fibroblast growth factor (FGF)-2, which are associated with new blood vessel formation in vitro.^421^ In addition, Id-1 expression is associated with intratumoral MVD, and there is a positive correlation between Id-1 overexpression and angiogenesis as well as poor clinical outcomes in OSCC.^422^

There is a significant correlation between tumor budding and shorter overall survival.^423,424^ Also, tumor budding correlates positively with lymph node metastasis.^425,426^ A study revealed that 33.9% of OSCC specimens displayed tumor budding, 58.9% of 56 OSCC patients have died, and the 5-year survival rate is 44.6%.^423^ However, no other clinicopathological factors are associated with tumor budding. Moreover, tumor budding is correlated with a rise in Snail expression and a tendency toward higher Twist expression. 46.4% of 56 OSCC specimens exhibit a positive expression of Snail, and 32.1% display a positive expression of Twist.^423^ Specifically, Snail is primarily located in cytoplasm and nuclei, whereas Twist is only present in a small number of nuclei. Expression of Snail and Twist are associated with lymph node metastasis in OSCC.^423^ However, well-differentiated OSCC expresses significantly less Twist, and there is no correlation between Snail or Twist expression and other clinicopathological factors. The overall survival rate of patients expressing Snail or Twist decreases dramatically.^423^ Taken together, tumor budding is strongly related to an unfavorable prognosis in patients with OSCC and correlates with the process of EMT.

Perineural invasion (PNI) may influence the progression of malignant cells and lead to poor prognosis. PNI has been detected in 17.4% of OSCC samples.^427^ In OSCC patients, miR-21/phosphatase and tensin homologs are abundant, and their dysregulation correlates with PNI and a poor prognosis.^428^ MMP-2 expressed by fibroblasts in the microenvironment of PNI is associated with a poorer prognosis in the treatment of OSCC and may be a contributing factor in OSCC PNI.^429^ Moreover, tumors with PNI have substantially higher levels of nerve growth factor (NGF) and tyrosine kinase than tumors without PNI (84% and 92%, respectively). PNI is associated with advanced carcinomas and worse DSS. Therefore, PNI in OSCC can be predicted by a high expression of NGF and tyrosine kinase A. The overexpression of PNI and NGF can also lead to pain in OSCC patients.^427^ Taken together, the expression of PNI and NGF is capable of determining the aggressiveness and prognosis of oral cancers in patients.^427^

Typically, prognosis has been correlated with the stage of the tumor. The 5-year survival rate for oral cancer patients is 64.4% overall, and 79.8%, 70.0%, 57.6%, and 53.9% for stages I–IV, respectively, with clinical stages II-IV having a reduced survival.^430^ In a research of 274 patients with oral malignancies, the survival rate among them after 12, 24, 36, 48, and 60 months is approximately 80%, 60%, 46%, 40%, and 39%, respectively.^431^ However, over 60% of oral carcinomas are detected in the advanced phases.^432^ In conclusion, the low survival rate obtained can be attributed primarily to the high proportion of OSCC cases diagnosed at an advanced stage.

#### Biological factors

Several biological indicators can be used to assess the progression of oral malignancies in patients. Accumulating evidence suggests a causal relationship between HPV and OSCC. Independent of cigarette smoking and alcohol abuse, HPV is linked to an elevated possibility of suffering from oral malignancies.^433^ This association applies to high-risk HPV samples, including subtypes 16, 18, 33, and 35.^434^ Over 80% of HPV^+^ OSCC may be due to HPV-16.^435^

HPV infection categorizes tumors into two distinct groups with varying prognoses and therapeutic implications.^436^ Generally, HPV^+^OSCC patients have a better treatment response, a higher two-year overall survival rate, a reduced disease progression risk and an improved prognosis, and lower death and recurrence rate than HPV^-^ patients.^437^ p16 is one of the most investigated prognostic biomarkers of OSCC. Of note, HPV^+^ and p16^+^ patients have a higher overall survival rate than HPV^-^ or HPV^+^ but p16^-^ patients.^438^ When p53 interacts with E6 encoded by carcinogenic types of HPV (such as HPV^-^16 and HPV^-^18), it is proteolyzed by ubiquitin-dependent proteases.^434^ There is a significant difference between the level of wild-type p53 in HPV^+^ neoplasms and the elevated possibility of p53 mutations in HPV^-^ tumors, which is related to a favorable prognosis for HPV^+^ OSSC patients.^53^

Additional OSCC prognostic biomarkers include G3BP1, B7-H6, and FAM3C. Patients with overexpressed G3BP1 mRNA exhibit a lower overall survival rate. In OSCC, mRNA and protein levels of G3BP1 are significantly higher than in normal tissues.^439^ G3BP1 has a direct relationship with Ki67 and an inverse relationship with Cleaved-caspase 3. The correlation between CD4^+^ T-cell infiltration and G3BP1 mRNA levels is positive. Enrichment analysis reveals that G3BP1 participates in helicase/catalytic/ATPase activity functions as well as spliceosome/RNA transport/cell cycle pathways and can be used as a biomarker to predict the prognosis of OSCC.^440^ Moreover, B7-H6 is identified as a distinct prognostic factor in OSCC involving DFS and CSS. OSCC tissues express significantly more B7-H6 protein than normal oral mucosa. B7-H6 expression correlates with differentiation; OSCC patients with less B7-H6 expression or more differentiated tumor tissue may have a better prognosis.^441^ Family with sequence similarity 3 member C (FAM3C) is an additional prognostic indicator that is essential for EMT. Immunohistochemical staining of OSCC samples with FAM3C, EMT markers, CSC markers, and co-inhibitory immune checkpoints is utilized to evaluate FAM3C levels and pathological features of OSCC. Compared to healthy mucosa and epithelial dysplasia, the level of FAM3C in OSCC specimens increases, and patients with a higher FAM3C expression are more likely to have a poor prognosis.^442^ In addition, the expression of FAM3C correlates positively with immune checkpoints such as PD-L1, VISTA, and B7-H4, the EMT marker Slug, and the CSC markers SOX2 and ALDH1.^442^

## Conclusion and perspectives

OSCC is typically associated with oral mucosa and a variety of risk factors. To mitigate risks, it is now understood that electronic cigarettes must be regulated similarly to traditional cigarettes.^443,444^ It is also necessary to limit the consumption of alcohol^445^ and BQ.^446^ Given that HPV is an influential risk factor for OSCC,^447^ HPV vaccinations should be promoted globally.^448^ However, there is insufficient molecular evidence to support the hypothesis that HPV^+^ OSCC is driven by HPV, as HPV is not inherently an indicator of a biologically active virus.^449^ Besides, OSCC risk factors include Epstein–Barr virus (EBV), which makes early diagnosis of OSCC patients essential.^450^

Due to these risk factors, OPMDs may develop prior to the onset of OSCC. To better stratify patients and follow their risk of malignancy, pathology assessments of OPMDs must go beyond subjective evaluation and be standardized.^451^ Furthermore, additional data from epidemiologic studies are required to elucidate the population of patients at current and future risk for OPMDs and subsequently the progression of OSCC.^452^ Future research on the incidence of OPMDs and OSCC will necessitate advances in molecular biology and genetics to uncover more distinct indicators, as interventional strategies based solely on histopathology are insufficient.^453^ In addition, more exploration into the aberrant metabolism of OPMDs and OSCC may shed new light on their pathogenesis.^109,454^

Through early diagnosis, oral cancer survival rates could reach up to 80%-90%.^455–460^ It will be feasible to make more sensitive and specific diagnoses of premalignancy and cancer through the development and rigorous testing of new diagnostic tools.^461–463^ Specifically, accelerated advancements in artificial intelligence (AI) hold promise for mass oral cancer screening. Currently, research is being conducted to develop AI-based technologies for the identification of oral malignancies with improved sensitivity and specificity, and in the future, the use of AI-based mobile applications will be advantageous for both frontline healthcare workers and the general public.^464–468^ These technological advances may allow for the early detection and management of suspicious lesions.^469,470^ Further explorations into the mechanisms of oncogenesis will assist and promote the accuracy of early diagnosis. Through the availability of a vast amount of information from transcriptomics, genomics, proteomics, epigenomics, and metabolomics, high-throughput sequencing technology will enable the development of novel therapeutic approaches for the treatment of OSCC. Furthermore, nanomedicine will provide efficient OSCC therapies by generating multiple synergetic therapeutics.^471–479^ Already, oral cancer treatment has undergone a substantial transformation, leading to improved patient outcomes.^480^ A multidisciplinary team will be required to manage these tumors, consisting not only of surgeons and oncologists, but also of specialists evaluating the nutritional, mental, social, and oral status of the individuals before, during, and after treatment.^481^
